# Folic acid prevents inner hair cell degeneration via genomic stability

**DOI:** 10.1038/s41420-025-02880-4

**Published:** 2025-12-02

**Authors:** Ruijie Cai, Xiaotong Ma, Jiawen Lu, Hongchao Liu, Meijian Wang, Ziquan Wang, Qinyan Xi, Hao Wu, Zhihua Zhang, Huihui Liu

**Affiliations:** 1 Department of Otolaryngology-Head and Neck Surgery, Shanghai Ninth People’s Hospital, Shanghai Jiao Tong University School of Medicine, Shanghai, China; 2 Ear Institute, Shanghai Jiao Tong University School of Medicine, Shanghai, China; 3 Shanghai Key Laboratory of Translational Medicine on Ear and Nose Diseases, Shanghai, China; 4 Shanghai Jiao Tong University School of Medicine, Shanghai, China

**Keywords:** Auditory system, Hair cell

## Abstract

Damage to inner hair cells (IHCs) is a leading cause of hearing loss, typically initiating at the base region of the basilar membrane. However, the mechanisms and preventative strategies for IHC damage remain to be elucidated. This study revealed that IHCs in the low-frequency region exhibit a significantly faster calcium clearance rate than high-frequency IHCs. This difference is associated with different PMCA1 expression. We then generated an IHC-specific *Pmca1* knockout mouse model (*Pmca1* CKO) exhibiting profound hearing loss and IHC death. Using single-cell RNA-seq analysis, we found that the differentially expressed genes (DEGs) were related to tetrahydrofolate biosynthesis, DNA damage, and DNA repair dysfunction. We therefore treated *Pmca1* CKO mice with folic acid and found that it protected IHCs by reducing γ-H2A.X levels. In addition, we found that folic acid protected IHCs from noise-induced damage. Overall, our findings suggest that disrupted calcium homeostasis plays a role in IHC damage and that folic acid may be a promising therapeutic agent for protecting hair cells.

## Introduction

Hair cell loss is a common pathological feature of acquired sensorineural hearing disorders in humans, including presbycusis [1–4]. Since mammalian hair cells lack regenerative capacity, thus protecting hair cells from degeneration was a potential therapy strategy. Numerous risk factors for hair cell damage can accumulate over a lifetime, such as noise exposure, the aging process, genetic mutations, and ototoxic drugs [5]. Therefore, one of the goals of the current research is to develop novel drug candidates to protect hair cells from degeneration. However, no drugs have yet been approved by the US Food and Drug Administration (FDA) for protecting hair cell loss. A deeper understanding of the mechanisms underlying hair cell degeneration is therefore essential and holds significant potential for identifying effective therapies to prevent hearing loss.

The long-coiled structure of the cochlea is arranged tonotopically, and the vulnerability of hair cell degeneration was quite variable along the tonotopic axis. The high-frequency region of the cochlea is particularly vulnerable to damage compared to the low-frequency region. In conditions such as noise-induced, age-related and ototoxic drug-induced hearing loss, hair cell and neuronal loss typically begins at the base of cochlear and progresses towards the apex [6–9]. Inner hair cells (IHCs), which are the primary sensory receptors responsible for transmitting auditory signals to the brain, exhibit regional differences in vulnerability. Investigating these differences could provide valuable insights into the mechanisms of IHC degeneration. As with other neurons, calcium ions are important for sustaining IHC function, and the disruption of intracellular Ca^2+^ homeostasis could account for noise- and drug-induced hearing loss and presbycusis [10, 11]. Intracellular Ca^2+^ levels are regulated by a complex interplay of calcium channels, endogenous Ca^2+^-binding buffers, Ca^2+^-ATPases, and exchangers [12, 13]. Among these, the plasma membrane Ca^2+^ ATPase (PMCA) is essential for fine-tuning intracellular Ca^2+^ concentrations and maintaining neuronal Ca^2+^ homeostasis [14]. For example, mutations in the PMCA2a pump in outer hair cells have been shown to cause deafness in mice [15–17], and the expression of PMCA is associated with neuronal dysfunction in aging and neurodegenerative disorders. Disruption of Ca^2+^ homeostasis can trigger pathological conditions, including elevated reactive oxygen species (ROS) levels and reduced ATP production, ultimately leading to neuronal degeneration [18–20]. While oxidative stress is widely recognized as a key factor in noise-, drug-, and age-related hearing loss, the specific biological properties of IHCs relating to Ca^2+^ homeostasis under these conditions remain poorly understood, partly due to the lack of suitable animal models.

Although the precise molecular mechanisms driving neuronal cell loss remain inconsensus, there is growing evidence to suggest that genomic DNA damage and the subsequent cellular responses it triggers play a critical role in promoting apoptosis and neurodegenerative diseases [21–23]. For example, motor neuron death has been associated with the abnormal persistence of R-loops, which cause DNA damage [24]. Moreover, neurodegenerative diseases and aging are often associated with elevated ROS levels and other intracellular damaging agents that compromise genomic integrity [25, 26]. As terminally differentiated cells, hair cells must deal with DNA lesions throughout their lifespan. Dysfunction in DNA repair is common in neurodegenerative diseases and eventually contributes to the neuronal death [27]. Therefore, accurate and timely DNA repair processes are essential for maintaining genomic stability and ensuring normal cellular function. Understanding the precise mechanisms of hair cell damage therefore offers a valuable opportunity to develop novel and effective therapeutic interventions.

In this study, we first investigated the functional differences between IHCs in the low- and high-frequency regions of the cochlea. Our findings suggest that the rate of calcium extrusion may underlie the differential vulnerability of IHCs degeneration, highlighting the importance of calcium homeostasis. To further explore this, we generated a hair cell-specific knockout mouse model of *Pmca1*, demonstrating that disrupted calcium homeostasis leads to IHC degeneration and eventual cell death. This *Pmca1* CKO mouse model serves as a valuable tool for studying IHC degeneration. Transcriptomic analysis of IHCs from *Pmca1* CKO mice revealed alterations in key molecular pathways, including DNA damage/repair and tetrahydrofolate biosynthesis. Based on these findings, we evaluated the therapeutic potential of folic acid, a known promoter of DNA repair [28, 29], in protecting IHCs from damage. We further demonstrated that folic acid mitigates noise-induced hearing loss by reducing DNA damage. Taken together, this study provides new insights into the mechanisms of IHC damage caused by disrupted calcium homeostasis, suggesting that enhancing oxidative DNA repair may represent a promising therapeutic strategy for acquired sensorineural hearing loss.

## Results

### Frequency-dependent variation in the exocytosis with similar calcium influx

Inner hair cells exhibit tonotopic susceptibility to dysfunction or death, such as ototoxicity [30], noise exposure [31], and aging [3], which is a common reason for acquired sensorineural hearing loss. To investigate the mechanisms underlying this regional vulnerability, we conducted electrophysiological experiments on IHCs from different cochlear regions to elucidate potential physiological differences.

**Fig. 1:**
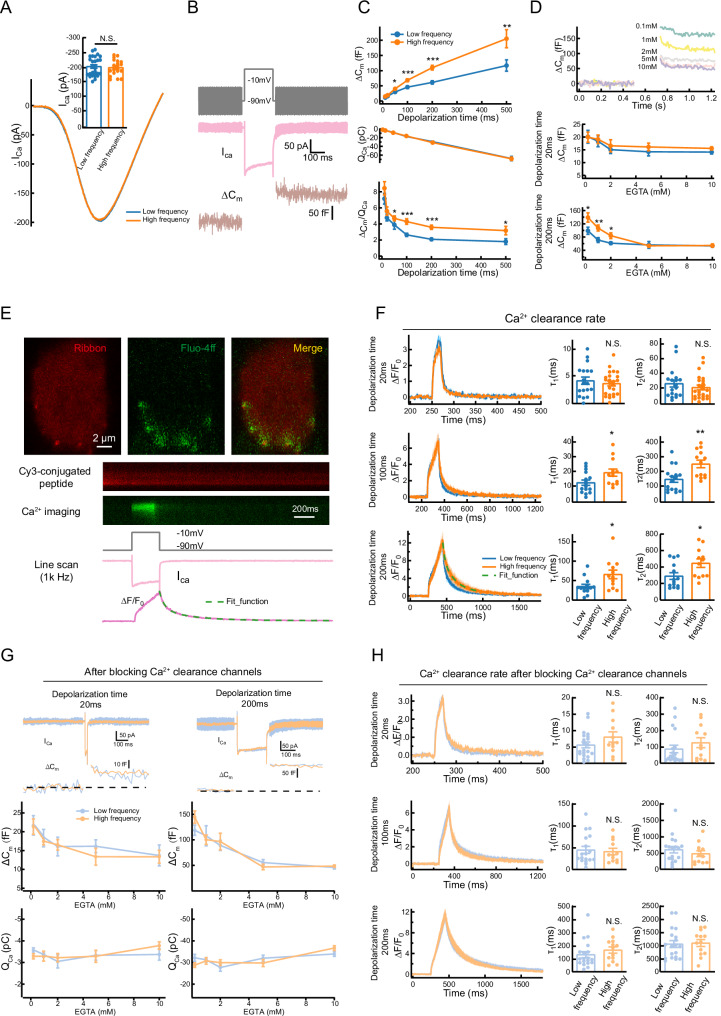
Physiological properties include calcium influx, calcium-dependent exocytosis and calcium clearance rate in low-and high-frequency IHCs. **A** Representative traces of Ca^2+^ currents (I_Ca_) traces from different frequency regions IHCs and quantitative analysis of I_Ca_ amplitude. **B** Representative stimulus protocol (top), I_Ca_ trace (middle) and corresponding membrane capacitance (ΔC_m_) traces (bottom) were recorded. **C** ΔC_m_ (top), calcium influx (Q_Ca_, middle) and ΔC_m_/Q_Ca_ (bottom) evoked by depolarizing pulses of varying durations in IHCs from different frequency regions. **D** Representative whole-cell capacitance measurements in IHCs with different concentrations of EGTA (0.1–10 mM) at a depolarization time of 200 ms (top). The ΔC_m_ at depolarization times of 20 ms (middle) and 200 ms (bottom) with different EGTA concentrations were quantified. **E** Two-photon imaging of calcium signals in IHCs. Top: Images showing calcium signals (green) and ribbon structures (red). Bottom: The stimulus protocol and quantification of the fluorescence intensity (ΔF/F₀) of calcium signals around individual ribbons. The decay of calcium signals was fitted to a single-exponential model to estimate the decay constant (τ). **F** Representative time-series data of ΔF/F_0_ with different depolarization durations (20 ms, 100 ms and 200 ms) and decay constants (τ1 and τ2) in low and high frequency IHCs. **G** ΔC_m_ in IHCs during 20 ms (left) and 200 ms (right) depolarizations at different EGTA concentrations (0.1–10 mM) after blocking Ca²⁺ extrusion. **H** Representative time-series data of ΔF/F₀ (left) and summary data of decay constants (τ₁ and τ₂, right) in response to different depolarization durations (20, 100, and 200 ms) after blocking Ca²⁺ extrusion in IHCs. Statistical analysis by two-side unpaired *t* test or Mann-Whitney test with significance indicated and two-way ANOVA followed by the Bonferroni post hoc test with significance indicated. All data, the number of data, statistical test used and *p* values can be found in the source data file. N.S., not significant, *p < 0.05; **p < 0.01; ***p < 0.001.

Using whole-cell patch-clamp recordings, we examined calcium influx (I_Ca_) in IHCs from specific frequency regions of the cochlea (Supplemental Fig. 1A). The amplitude of I_Ca_ was comparable across IHCs from different cochlear locations (Fig. 1A), consistent with previous findings [32]. We next used nonstationary noise analysis of Ca^2+^ tail currents to estimate the total number of Ca^2+^ channels per hair cell [33], and found that the IHCs located in the high-frequency region possess more Ca^2+^ channels but the size of single-channel current was much smaller (Supplemental Fig. 1B). In addition, the Ca^2+^ current amplitude remained comparable when the Ca^2+^ channel agonist BayK 8644 was applied (Supplemental Fig. 1C), indicating similar Ca^2+^ channel open probabilities across regions (Supplemental Fig. 1B). These results suggest that calcium influx in IHCs is comparable under similar depolarization conditions, regardless of their tonotopic position.

To determine whether Ca^2+^-dependent exocytosis varies with frequency position, we measured changes in membrane capacitance (ΔC_m_) in response to depolarizing stimuli of varying durations (Fig. 1B, C). IHCs from the high-frequency region revealed a significantly enhanced vesicle release with a similar calcium influx. Moreover, the Ca^2+^ efficiency of triggering exocytosis, assessed with the ratio of ΔC_m_/Q_Ca_, is much more efficient for stimulation from 50 to 500 ms (Fig. 1C). Previous study has demonstrated that this position-dependent difference was not related to the expression level of the Ca^2+^ sensor (otoferlin) nor the colocalization between Ca^2+^ channels and release sites [34]. We therefore hypothesize that elevated cytosolic Ca^2+^ concentrations near the ribbon synapse during IHC depolarization may facilitate exocytotic responses. To test this, we examined vesicle release kinetics in the presence of varying concentrations of EGTA, a Ca^2+^ chelator. While EGTA did not affect exocytosis during short depolarizations, high-frequency IHCs exhibited significantly greater exocytosis than low-frequency IHCs during longer depolarizations (200 ms) at lower EGTA concentrations. This difference diminished at higher EGTA concentrations (Fig. 1D).

Our previous study indicated that slow calcium clearance around the presynaptic ribbon could lead to the elevation of [Ca]i [35]. To explore this further, we measured the cytosolic Ca^2+^ clearance rate near the active zone of IHCs following depolarization (Fig. 1E, F). We loaded IHCs with Cy3-conjugated Ribeye-binding peptide to locate the ribbons and a line scan (1 kHz) across the center of the ribbon to record the fluorescence change of Fluo-4FF (Fig. 1E) [35, 36]. The time constant fitted by a double exponential function during the decay phase of the Ca^2+^ transient represents the rate of cytosolic Ca^2+^ clearance [37, 38]. Although the decaying component was similar between the two region IHCs under short-depolarization time, IHCs of the high-frequency region exhibited a slower decay time constant, indicating that the Ca^2+^ clearance capability of the IHCs at the high-frequency region was much weaker (Fig. 1F).

Next, we wanted to investigate whether and how the Ca^2+^ clearance could affect exocytosis, we blocked Ca^2+^ extrusion channels with pharmacological treatments [39]. Blocking these channels reduced the differences in exocytosis between high- and low-frequency IHCs, particularly at lower EGTA concentrations and during both short and long depolarizations (Fig. 1G). Similar results were observed when measuring Ca^2+^ clearance rates following channel blockade (Fig. 1H). Together, these findings suggest that high-frequency IHCs are more sensitive to changes in Ca^2+^ clearance rates than low-frequency IHCs. Therefore, we propose that the heightened susceptibility of basal (high-frequency) IHCs to damage may be due to slower Ca^2+^ clearance.

### PMCA1 is the main calcium clearance channel and the *Pmca1* CKO mice showed pronounced hearing loss

**Fig. 2:**
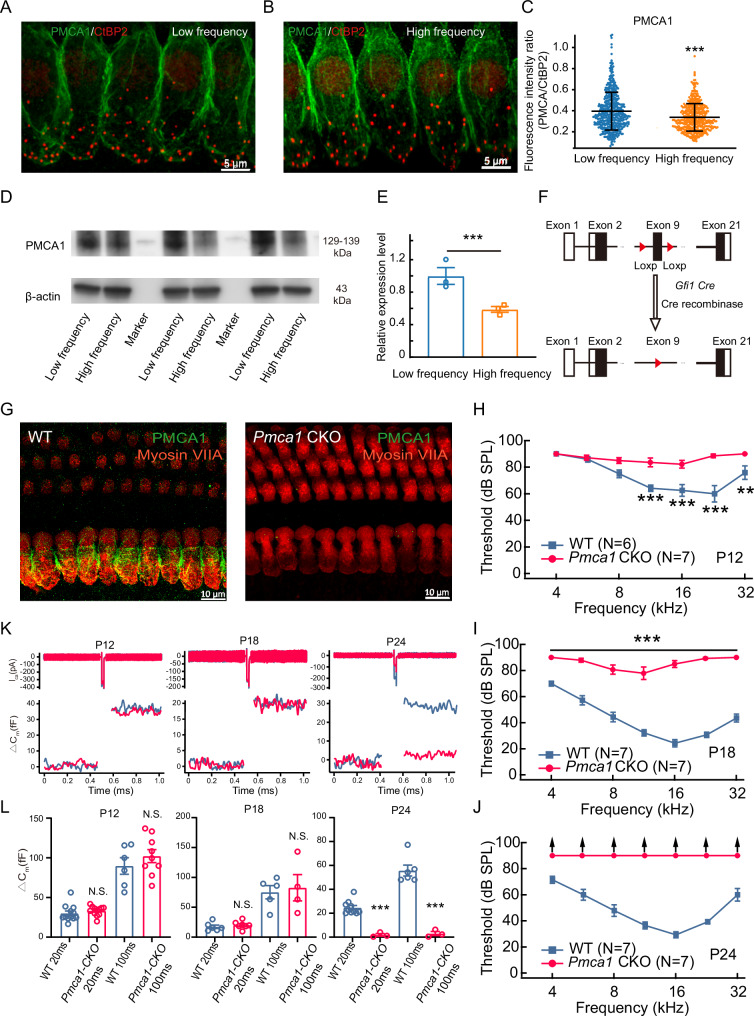
PMCA1 channel expression level along the cochlea and auditory function in *Pmca1* CKO mice. **A**–**C** Gradient expression of PMCA1 in IHCs. Immunofluorescence images of PMCA1 (green) and ribbon synapses (red) in the low-frequency (**A**), high-frequency (**B**) regions, with corresponding quantification of the PMCA1 fluorescence intensity ratio (**C**). **D**–**E** The expression of PMCA1 proteins from apex- and base- basilar membrane was quantified by western blot. Three samples (2 cochlear from same mouse pooled for each sample) were used for immunoblot analysis. **F** Strategy for conditional knockout of *Pmca1* in hair cells, depicting LoxP sites inserted to flank exon 9. **G** Cochlear whole-mounts immunostaining with anti-Myosin VIIA antibody (red) to label hair cells and stained with anti-PMCA1 antibody to detect PMCA1 protein (green). **H**–**J** ABR thresholds are elevated in *Pmca1* CKO mice, compared with WT controls. Progression of hearing loss is seen in *Pmca1* CKO mice from P12, P18 and P24. **K**, **L** Representative capacitance traces (**K**) and quantification of ΔC_m_ (**L**) evoked by 20 ms and 200 ms depolarizations in IHCs from mice of different ages, showing the functional consequence of impaired calcium clearance. Statistical analysis by two-side unpaired *t* test or Mann-Whitney test with significance indicated and two-way ANOVA followed by the Bonferroni post hoc test with significance indicated. All data, the number of data, statistical test used and *p* values can be found in the source data file. N.S., not significant, *p < 0.05; **p < 0.01; ***p < 0.001.

To determine which channel expression was associated with a longitudinal gradient in IHCs, we compared the fluorescence intensity around the presynaptic ribbons (integrated within a 0.5-μm radius around the center of mass of CtBP2 fluorescence, Fig. 2A, B) [35]. Our analysis revealed a significant decrease in PMCA1 expression from the apex to the base of the basilar membrane (Fig. 2C). In contrast, the expression levels of other calcium-handling proteins, including the sodium-calcium exchanger (NCX), sarco/endoplasmic reticulum Ca^2+^-ATPase (SERCA), and mitochondrial calcium uniporter (MCU), showed no significant variation along the tonotopic axis (Supplemental Fig. 2A-C). The gradient in PMCA1 expression was further confirmed by Western blot analysis, which revealed different expression patterns between low- and high-frequency regions (Fig. 2D). These findings suggest that PMCA1 may play a critical role in the regional susceptibility of IHCs to degeneration.

To rule out the effects of PMCA1 in the hair cells and hearing, we generated a hair cell *Pmca1* conditional knockout mouse line (Fig. 2E-G). We crossed mice (*Pmca1*^*Loxp/Loxp*^) with a transgenic Cre driver mouse that expresses Cre under the *Gfi1* promoter [40], which is active in hair cells [41, 42], to generate *Pmca1* CKO mice. Immunofluorescent staining revealed an absence of PMCA1 in *Pmca1* CKO mice IHCs (Fig. 2F, G). Notably, the deletion of PMCA1 did not result in any overt morphological changes in the hair bundles (Supplemental Fig. 3).

Auditory brainstem response (ABR) measurements, which reflect the electrical activity of cochlear ganglion neurons and central auditory nuclei in response to sound stimulation [43], revealed profound hearing impairment in *Pmca1* CKO mice. Hearing thresholds were significantly elevated at postnatal days 12 (P12) and 18 (P18) across frequencies ranging from 8.0 kHz to 22.6 kHz. By P24, ABR waveforms were completely absent, indicating severe IHC degeneration in *Pmca1* CKO mice (Fig. 2H-J). These results demonstrated that PMCA1 is essential for maintaining normal auditory function. In subsequent experiments, we explored the potential mechanisms underlying this hearing impairment.

To test whether presynaptic exocytosis dysfunction accounted for the elevated ABR threshold, we recorded exocytosis in response to depolarizing stimuli (Fig. 2K, L). Exocytosis was significantly attenuated in *Pmca1* CKO IHCs at P24, indicating impaired IHC function. However, evoked ΔCm in IHCs was comparable between wild-type (WT) and *Pmca1 CKO* mice at P12 and P18. The elevated ABR threshold may therefore result from unsynchronized firing of the auditory nerve [39]. Therefore, the *Pmca1* CKO mouse model could be used to study the mechanism of IHC damage.

### Calcium homeostatic imbalance leads to progressive inner hair cell damage

**Fig. 3:**
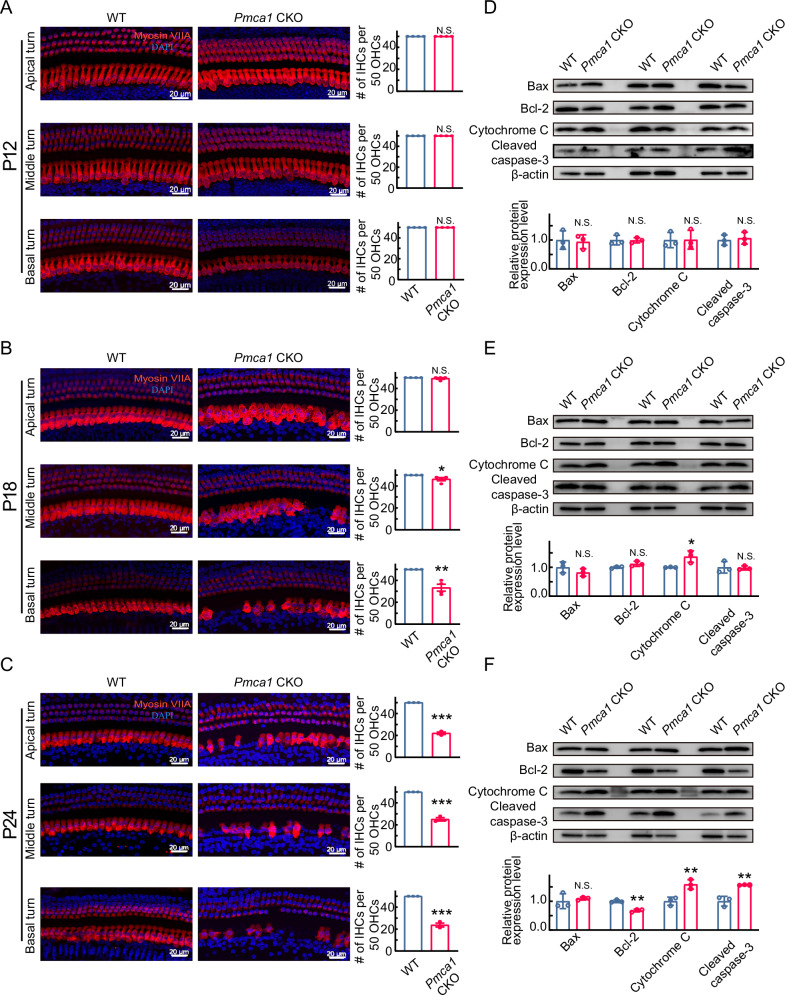
The survival of hair cells and expression level of apoptosis-related proteins in *Pmca1* CKO mice. **A**–**C** Representative confocal images of hair cells in *Pmca1* CKO and control mice at P12 (**A**), P18 (**B**) and P24 (**C**) from the apex (top), middle (middle) and base (bottom). Hair cells were labeled with an anti-Myosin VIIa antibody (red), and nuclei were counterstained with DAPI (blue). **D**–**F** Quantification of the expression of apoptosis-related proteins at P12 (**D**), P18 (**E**) and P24 (**F**) in *Pmca1* CKO and control mice was performed by immunoblotting. β-Actin was used as the internal control. Three samples (two cochlear samples pooled from the same mouse for each sample) were used for immunoblot analysis. The expression of cytochrome C and cleaved caspase-3 increased, while Bcl-2 decreased, at P24, with comparable levels of these proteins at P12 and P18 (excluding cytochrome C at P18). Statistical analysis by two-side unpaired *t* test or Mann-Whitney test with significance indicated. All data, the number of data, statistical test used and *p* values can be found in the source data file. N.S., not significant, *p < 0.05; **p < 0.01; ***p < 0.001.

To investigate the mechanisms underlying the profound hearing loss in *Pmca1* CKO mice, we first examined the morphology of the basilar membrane at P12, P18, and P24. At P12, *Pmca1* CKO mice exhibited normal hair cell populations (Fig. 3A). However, by P18, IHC loss was observed, initially localized to the basal region of the cochlea. This loss had progressed further by P24, extending towards the apical region (Fig. 3B, C). We next assessed whether the number of ribbon synapses was affected in surviving IHCs following *Pmca1* deletion. At P12, the number of ribbon synapses in *Pmca1* CKO mice was comparable to that in control mice. However, by P18 and P24, a significant reduction in ribbon synapse counts was observed, progressing from the base to the apex of the cochlea (Supplemental Fig. 4A-C). Moreover, whole-cell patch-clamp recordings revealed a significant reduction in the peak amplitude of Ca^2+^ currents (I_Ca_) in *Pmca1* CKO mice at P18 and P24 compared to controls, which is consistent with IHC degeneration (Supplemental Fig. 4D-F). In contrast, no significant differences in I_Ca_ were observed at P12. These results indicate that IHC degeneration and death occur after hearing onset, likely due to the sound-evoked activity of IHCs. These findings suggest that calcium homeostatic imbalance contributes to IHC damage and eventual cell loss following hearing onset, ruling out IHC dysfunction/ loss as the primary cause of the observed hearing phenotype.

Next, we investigated how PMCA1 deletion led to the degeneration/death of IHCs. First, we measured a series of factors involved in the IHCs degeneration process [8, 44–46], including Bcl-2, Bax, Cleaved caspase-3, and Cytochrome-c. Their expression was comparable between *Pmca1* CKO and WT mice at P12, whereas the expression of Bax/Bcl-2, cleaved caspase-3, and cytochrome C was significantly up-regulated in the *Pmca1* CKO cochlea at P18 and P24 (Fig. 3D-F). These results suggest that calcium homeostatic imbalance eventually led to IHCs apoptosis, which is a similar pathological process of acquired hearing loss; however, the signaling networks that link calcium homeostatic imbalance to apoptosis remain unclear.

### Transcriptional changes in IHCs of *Pmca1* CKO mice implicate DNA damage

**Fig. 4:**
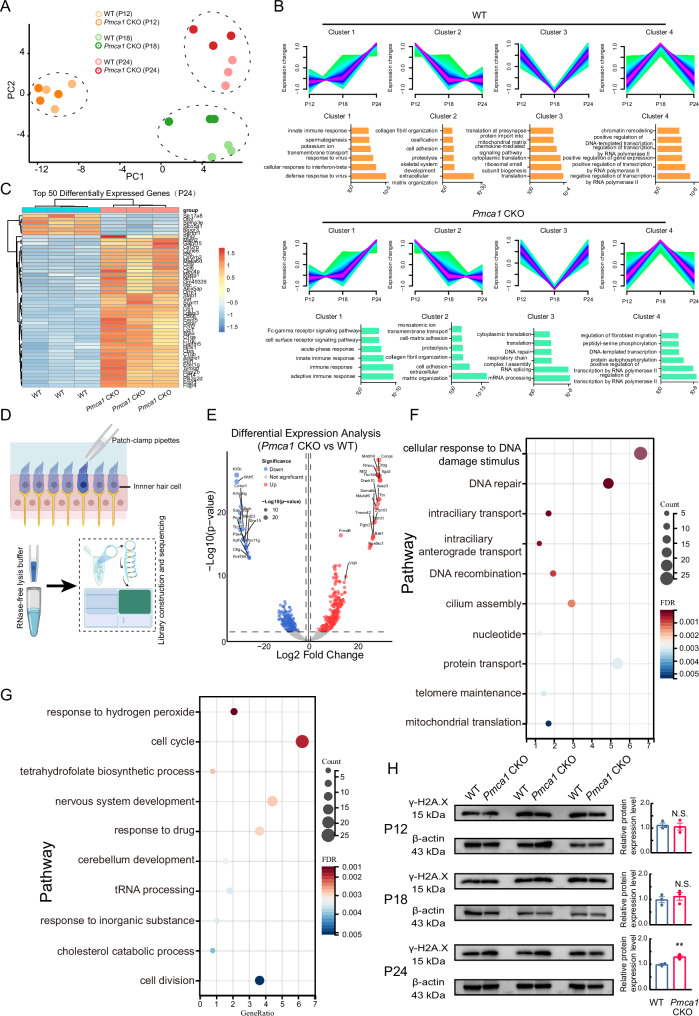
Folic acid treatment prevents hair cells from damage in *Pmca1* CKO mice. **A** Principal component analysis (PCA) of the inner hair cell (IHC) transcriptomes of WT and *Pmca1* CKO mice of each age group. **B** The corresponding gene expression trajectories and representative Gene Ontology (GO) terms. **C** A heatmap showing the top 50 differentially expressed genes in IHC at P24. **D** Diagram showing the inner hair cell RNA sequencing procedure. **E** A representative volcano plot showing the DEGs between WT and *Pmca1* CKO mice. **F**, **G** Representative GO enrichment analysis for DEGs (**F:** up-regulated; **G**: down-regulated) between wild-type (WT) mice and *Pmca1* CKO mice. **H** Quantification of γ-H2A.X expression in *Pmca1* CKO and control mice by western blot at P12, P18 and P24. Three samples (two cochleae from the same mouse, pooled for each sample) were used for immunoblot analysis. Statistical analysis by two-side unpaired *t* test with significance indicated. All data, the number of data, statistical test used and *p* values can be found in the source data file. N.S., not significant, *p < 0.05; **p < 0.01; ***p < 0.001.

To characterize the transcriptomic profiles changes underlying biological properties of *Pmca1* CKO mice, we first performed RNA sequencing (RNA-seq) on the cochlear basilar membrane. The gene expression profile showed developmental and group (expect P12) discrimination (Fig. 4A).

We then classified the expressed genes into four clusters with - gradual increase (cluster 1), gradual decrease (cluster 2), biphasic decrease followed by increase (cluster 3) and biphasic increase followed by decrease (cluster 4) along different developmental stages (Fig. 4B). Pathway analysis revealed differential enrichment between WT and *Pmca1* CKO mice, particularly within clusters 1 and 3 of the *Pmca1* CKO group. These clusters encompassed pathways involved in immune response, mRNA processing, DNA repair, and other functions (Fig. 4B). These results support the consensus that IHCs damage results from calcium imbalance. The differentially expressed genes (DEGs) between WT and *Pmca1* CKO mice are presented in Fig. 4C and Supplementary Fig. 5A, B.

We further assessed differentially expressed genes in IHCs from the two groups. We harvested inner hair cells from mice aged 1 month, including WT and *Pmca1* CKO mice (Fig. 4D). We collected and analyzed 10 IHCs from WT mice, 12 IHCs from *Pmca1* CKO mice, and produced 24.3 million sequence reads per library, of which ~ 80% were mapped to the mm10 genome, and an average detection of ~9499 genes per IHC (WT: 9023.57 ± 1234.2 genes, *Pmca1* CKO: 10054.50 ± 1456.8 genes). As shown in the Supplemental Fig. 5C, our analysis confirmed that the transcriptome data displayed a high degree of specificity to IHCs (e.g., *Otof* and *Slc17a8*). We found that 355 genes were downregulated and 1143 genes were significantly upregulated in *Pmca1* CKO mice. Gene set enrichment analysis of Gene Ontology (GO) biological processes revealed significant enrichment of pathways related to DNA damage, DNA repair, and DNA recombination in *Pmca1* CKO mice, indicating that calcium homeostatic imbalance triggered DNA damage and repair response programs. The cell cycle, tetrahydrofolate biosynthetic process, and nervous system development pathway were significantly decreased in *Pmca1* CKO mice (Fig. 4F, G). As a marker of DNA damage and repair, γ-H2A.X plays an important role in the recognition and repair of DNA double-stranded breaks. Western blot analysis confirmed elevated levels of γ-H2A.X in *Pmca1* CKO mice (Fig. 4H), which further supports the hypothesis that calcium dysregulation induces DNA damage and activates repair mechanisms. Taken together, these findings suggest that calcium homeostatic imbalance upregulates DNA damage and repair response pathways, ultimately contributing to IHC damage.

### Folic acid could protect IHCs against damage by enhancing DNA repair

**Fig. 5:**
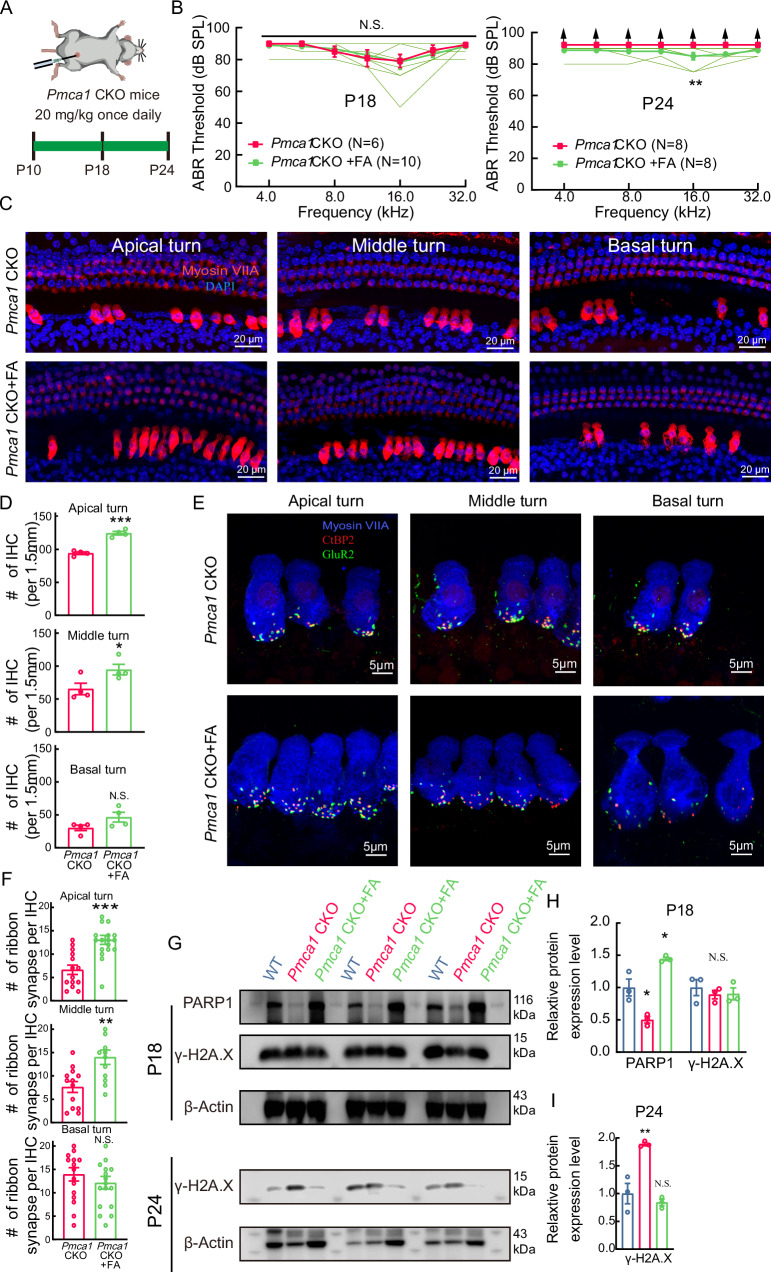
Folic acid treatment prevents hair cells from damage in *Pmca1* CKO mice. **A** Experimental design showing auditory tests and folic acid treatment. From postnatal day 10 (P10), the mice received folic acid intraperitoneally once a day and hearing threshold was measured at P18 and P24. **B** ABR thresholds in untreated *Pmca1* CKO mice (*Pmca1* CKO, red) and *Pmca1* CKO mice treated with folic acid (*Pmca1* CKO + FA, green) at P18 and P24. Individual data (thin green lines) demonstrate that folic acid treatment partially restored hearing sensitivity, particularly at 16.0 kHz. **C**, **D** Representative confocal images of hair cells in untreated (left) and treated (right) *Pmca1* CKO mice from the apex, middle and base turns at P24. Hair cells were labeled with anti-Myosin VIIa (red) and nuclei with DAPI (blue). Folic acid treatment significantly preserved the number of inner hair cells (IHCs). **E**, **F** Representative confocal images of ribbon synapses in the apical, middle, and basal turns of untreated (**E**) and folic acid-treated (**F**) *Pmca1* CKO mice at P24. Tissues were immunostained for Myosin VIIa (hair cells, blue), CtBP2 (presynaptic ribbons, red), and GluR2 (postsynaptic receptors, green). Folic acid treatment prevented the loss of ribbon synapses. **G**−**I** The expression of DNA repair/damage-related proteins at P18 and P24 from *Pmca1* CKO and treated *Pmca1* CKO mice was quantified by western blot. Three samples (two cochlear samples pooled from the same mouse for each sample) were used for immunoblot analysis. PARP1 expression was decreased in *Pmca1* CKO mice and increased after treatment in *Pmca1* CKO mice at P18 (**H**). γ-H2A.X expression was comparable in all groups at P18 (**H**). However, at P24, the expression of γ-H2A.X was significantly lower in the treated *Pmca1* CKO mice than in the untreated *Pmca1* CKO mice (**I**). Statistical analysis by two-side unpaired *t* test or Mann-Whitney test with significance indicated and two-way ANOVA followed by the Bonferroni post hoc test with significance indicated. All data, the number of data, statistical test used and *p* values can be found in the source data file. N.S., not significant, *p < 0.05; **p < 0.01; ***p < 0.001.

Folic acid (FA), which is involved in DNA synthesis and repair [47, 48], was administered (*i.p*., 20 mg/kg, every other day) to *Pmca1* CKO mice at P10 for 14 days (Fig. 5A). There was no significant difference in the ABR threshold at P18 between treated (*Pmca1* CKO + FA) and untreated *Pmca1* CKO mice. However, the ABR wave was still visible at P24 after injection of folic acid, indicating the protective function of folic acid in the hair cell of *Pmca1* CKO mice (Fig. 5B). To further evaluate the protective effect of folic acid, we then counted the number of hair cells, which were stained with DAPI (blue) and labeled with MyosinVIIa (red). The results suggest that folic acid significantly reduced IHC loss (Fig. 5C, D).

Ribbon synapses, which are located between IHCs and SGNs, are the most critical structures that promote rapid neurotransmitter release and sustained signal transmission in the IHCs. To explore the protective effect of folic acid against the loss of IHC ribbon synapses in *Pmca1* CKO mice, we quantified the number of paired ribbon synapses at P24. Presynaptic structures were labeled with CtBP2 (red), and postsynaptic structures were labeled with GluR2 (green). Regions in which CtBP2 and GluR2 (yellow) colocalized were identified as functional (paired) synapses. The results showed that treatment with folic acid markedly reduced the loss of paired synapses in the *Pmca1* CKO mice (Fig. 5E, F).

We hypothesized that folic acid treatment attenuates hair cell loss, at least in part, by inhibiting of DNA damage. Recent evidence has implicated poly (ADP-ribose) polymerase 1 (PARP1) in various DNA repair pathways and in maintaining genomic stability [49, 50]. PARP1 expression is decreased in IHCs of *Pmca1* CKO mice at P18; however, increases following folic acid treatment. Furthermore, the injection of folic acid inhibited the high expression of γ-H2A.X protein at P24 (Fig. 5G-I). These results indicate that folic acid can prevent IHC death in the *Pmca1* CKO mice.

### DNA damage in noise-induced hearing loss and enhanced DNA repair as a protective strategy

Noise-induced hearing loss (NIHL) is the most common form of non-hereditary sensorineural hearing loss, and severe noise exposure can damage cells in the inner ear, resulting in HC loss and elevated hearing thresholds. Calcium overload in sensory hair cells has been well documented after traumatic noise exposure [10]. Based on our findings, we wondered whether noise exposure could lead to DNA damage and whether enhanced DNA repair could alleviate the hearing threshold shifts.

We then evaluated the therapeutic efficacy of folic acid against noise-induced hearing loss by administering it intraperitoneally (*i.p*., 20 mg/kg daily) for 7 days before noise exposure. The hearing function of mice was tested by measuring the ABR at day 1 before and after noise exposure. The hearing threshold of NIHL mice treated with folic acid at day 1 (Treament_PND1) and 14 after noise exposure (Treament_PND14) was reduced compared to group without treatment of folic acid (PND1 and PND14), indicating a protection for their hearing.

**Fig. 6:**
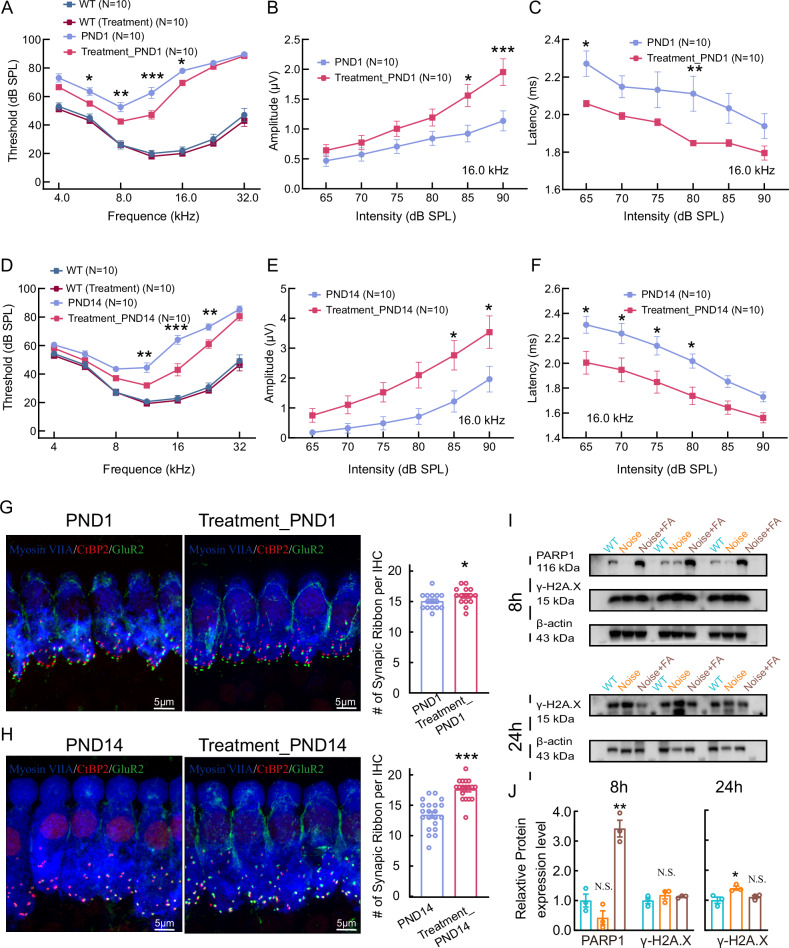
Folic acid treatment attenuates noise-induced hearing loss. **A**–**F** Folic acid treatment preserves auditory function after noise exposure. ABR thresholds and ABR wave I amplitudes were measured in wild-type mice with or without FA treatment before (pre-) and at 1 and 14 days post-exposure. Representative confocal images of ribbon synapses in mice with or without folic acid treatment at one day (**G**) and 14 days (**H**) post-exposure. Basilar membranes were stained with an anti-Myosin VIIA antibody (blue) to label hair cells, CtBP2 (red) to identify the presynapse and GluR2 (green) to identify the postsynapse. The number of ribbon synapses is increased in treated mice compared with untreated mice. **I**, **J** Quantification of the expression of DNA-repair/damage-related proteins at 8- and 24 h post-exposure in treated and untreated mice was performed by western blot. Three samples (two cochlear samples pooled from the same mouse for each sample) were used for immunoblot analysis. PARP1 expression was decreased in untreated mice and increased after treatment, while γ-H2A.X expression was comparable in all groups at 8 h after noise exposure. γ-H2A.X expression was significantly decreased in treated mice at 24 h post-noise exposure. Statistical analysis by two-side unpaired *t* test or Mann-Whitney test with significance indicated and two-way ANOVA followed by the Bonferroni post hoc test with significance indicated. All data, the number of data, statistical test used and *p* values can be found in the source data file. N.S., not significant, *p < 0.05; **p < 0.01; ***p < 0.001.

The first wave of ABR (Wave I) represents the summated activity of responding auditory afferent fibers. Similarly, at day 1 and 14 after noise exposure, the wave I amplitudes of ABRs were higher and the latency was shorter in mice treated with folic acid than the control group (Fig. 6A). By co-staining the presynaptic ribbons (CtBP2) and postsynaptic GluR2 of the organ of Corti before and after noise exposure, we found that the number of ribbon synapses was increased in mice treated with folic acid at day 1 and 14 after noise exposure (Fig. 6G, H).

Next, we investigated how folic acid protects the function of IHCs. First, we measured the expression levels of PARP1, which involved in the DNA repair process. At 8 hours (8 h) after noise exposure, the expression level of PARP1 was decreased in NIHL mice but increased in NIHL mice treated with folic acid compared to control. Additionally, at 24 h after noise exposure, the expression of γ-H2A.X was significantly increased in group without treatment of folic acid, while comparable in groups with the treatment of folic acid compared with the control group (Fig. 6I, J). These results provide strong evidence that folic acid is a potent otoprotectant against noise induced hearing loss through protecting DNA against damage.

## Discussion

A lack of appropriate animal models that reflect the key mechanisms of hair cell damage or death may be one reason why molecular targets for drug discovery have been difficult to identify. Due to the tonotopic susceptibility of the hair cells to noise exposure, ototoxic drugs, and aging, we compared the biophysical properties of IHCs across different frequency regions to identify vulnerability factors. Our findings revealed that IHCs in the high-frequency region exhibit a slower calcium clearance rate. Consequently, we hypothesize that the differential vulnerability of IHCs may be associated with variations in calcium homeostasis maintenance. To investigate the biological underpinnings of this phenomenon, we generated *Pmca1* CKO mice, which displayed progressive IHC damage, consistent with our hypothesis. Transcriptomic analysis of IHCs indicated that DNA damage might be the primary driver of IHC degeneration. To test this hypothesis, we administered folic acid to assess whether mitigating DNA damage could protect IHCs in *Pmca1* CKO mice. This protective effect was further validated in a noise-induced cochlear injury model. Collectively, our findings demonstrate, for the first time, that DNA damage prevention represents a promising strategy for preventing and treating acquired sensorineural hearing loss.

In our earlier work, we reported that CBA/CaJ and C57BL/6 J mice exhibited different phenotypes in synaptopathy after noise exposure due to the difference in Ca^2+^ clearance rate [35]. In this study, we showed that the tonotopic variation in exocytosis of IHCs is likely to result from a different function of calcium extrusion, which could be determined by the excessive Ca^2+^ around the ribbon synapse [39]. IHCs in the low-frequency region of the cochlea possess a faster calcium clearance rate, consistent with the finding that phase-locking occurs at low frequencies (<4 kHz) [46]. We thus inferred that the elevation of cytosolic Ca^2+^ concentration may be a major contributing factor to the high-frequency IHC damage during noise-, chemical exposure, and aging. We further revealed that the expression pattern of PMCA1 was decreased in the IHCs from the low to high-frequency region of the cochlear, which is similar to PMCA2 [51]. Although a tonotopic gradient in Ca^2+^ binding protein expression has been reported in IHCs [32], no IHCs death was found in the genetic disruption of parvalbumin-α, calbindin-D28k, and calretinin mice [52]. In contrast, conditional knockout of *Pmca1* could lead to gradual IHCs death, indicating that PMCA1 was the main component to maintaining calcium hemostasis of IHCs. Thus, we used *Pmca1* CKO mice, as a calcium imbalance animal model, to decipher the pathophysiology of hair cell damage, which may provide useful information for the treatment of [Ca]_i_ imbalance-related hair cell damage.

Our findings suggest that DNA damage plays a causal role in the pathological of IHCs damage of *Pmca1* CKO mice. The accumulation of DNA damage in neuronal cells either drives the loss of genome integrity or interferes with gene regulatory processes, thereby promoting the activation of cell death responses and consequent cell loss [53]. From the RNA-seq of IHCs, we observed that the downregulated differential expression genes were related to the cellular response to DNA damage stimulus, DNA repair, DNA recombination, et al., indicating DNA repair impairment and genomic instability in *Pmca1* CKO mice. DNA damage and repair could lead to changes in gene expression, cellular dysfunction, or even cell death if the damage is not repaired in a timely manner [54–57]. Thus, we inferred that intracellular Ca^2+^ overload contributes to challenging the genome integrity of IHCs and eventually leads to IHCs death. Moreover, in the nervous system, DNA damage and repair impairment could result in neurodegenerative disorders, such as amyotrophic lateral sclerosis [58], Alzheimer’s disease [59, 60], and Parkinson’s Disease [61]. Based on these observations, we hypothesized that DNA repair impairment and the accumulation of genome damage may be potential mechanisms of hair cell degeneration. As a consequence, we successfully prevented hair cell damage in *Pmca1*^*-\-*^ mice and alleviated noise-induced hearing loss using folic acid, which could increase DNA repair [29].

Numerous studies have identified key parameters in the pathogenesis of noise-, drug-, or aging-related hearing loss, including calcium overload, ATP depletion, and excessive oxidative stress [62, 63]. However, therapeutic strategies such as antioxidants, anti-inflammatory agents, calcium channel blockers, and kinase modulators have not yet been effectively translated into clinical applications for hearing loss prevention [64–66]. This study demonstrates that folic acid supplementation protects against hair cell death, suggesting its potential as a therapeutic agent for acquired hearing loss. Folic acid plays a critical role in DNA synthesis, repair, and methylation [67–69]. Previous studies have shown that folic acid supplementation can enhance neuronal function by improving circulating homocysteine levels, deoxythymidine triphosphate biosynthesis, and DNA methylation [70–74]. We further investigate the potential mechanisms and found that folic acid supplementation reduced hair cell apoptosis by modulating the expression of Bcl-2, Bax, and Caspase-3. Moreover, the DNA damage was alleviated may be due to the higher expression of PARP1 after folic acid supplementation. Despite the differences in etiology and pathophysiology of IHCs in *Pmca1*^*-\-*^ mice compared with other models, it can be stated that the interventions can be also feasible for NIHL and ARHL because they have some overlaps in the pathological process as well. Thus, targeting DNA damage and endogenous DNA repair mechanisms may hold promise for accelerating hair cell repair and functional recovery.

Our study highlights the clinical translational potential of folic acid in addressing age-related hearing loss (ARHL) and sudden hearing loss (SHL). In ARHL, oxidative stress [75–77], calcium dyshomeostasis [78], and DNA damage [79, 80] are the main drivers of IHC degeneration. Folic acid mitigates these processes by enhancing DNA repair [81], restoring calcium balance [73], and reducing oxidative stress [82], offering a promising therapeutic strategy. In the case of SHL, which is often associated with vascular compromise or metabolic dysfunction, folic acid’s role in improving DNA repair and reducing oxidative stress aligns with its potential to restore cochlear function [83]. Its safety, affordability, and established use in clinical settings make it an ideal candidate for immediate trials in ARHL and SHL populations [84]. Future research should focus on optimizing dosing and exploring synergistic effects with antioxidants to maximize its therapeutic efficacy, positioning folic acid as a cornerstone in hearing loss treatment.

Overall, this study demonstrates that calcium homeostasis imbalance leads to hair cell damage via DNA damage, and that folic acid mitigates hair cell loss and improves hearing function following noise exposure by alleviating DNA damage. Our findings underscore the therapeutic potential of enhancing DNA repair in hair cells and position folic acid as a promising clinical agent for the prevention and treatment of hearing loss.

## Method

### Animals

Wild-type (C57BL/6 J) and genotypic mice of both sexes were obtained from Shanghai Model Organisms Center, Inc., Shanghai, China. Mice were housed for the duration of these experiments in the animal care facility of the Ear Institute of Shanghai Ninth People’s Hospital, in affiliation with the Shanghai Jiao Tong University School of Medicine. The experimental protocol was approved by the University Committee of Laboratory Animals of Shanghai Ninth People’s Hospital (Approval No. SH9H-2022-A926-1) and followed the guidelines for the Care and Use of Laboratory Animals (8th edition), published by the National Institutes of Health (Bethesda, MD, USA). The experimental and control groups were housed in separate, unlabeled cages, and investigators were blinded during data acquisition and analysis.

### Hearing assessment

ABR measurements were conducted like that described in our previous study [35, 85]. Briefly, animals were anesthetized and all recordings were conducted in a sound-attenuating chamber. A customized TDT System 3 (Tucker-Davis Technologies Inc., Alachua, FL) was used for ABR recordings. Differentially recorded scalp potentials were bandpass filtered between 0.05 and 3 kHz over a 15 ms epoch. A total of 400 responses were averaged for each waveform for each stimulus condition.

### Immunohistochemistry staining

The cochleae were perfused with 4% paraformaldehyde immediately after dissection and fixed for 30 min. Thereafter, the basilar membrane was dissected then permeabilized and blocked for 60 min in 0.5% (v/v) Triton X-100 and 4% (w/v) BSA/PBS at room temperature before incubation with the following primary antibodies: rabbit polyclonal anti-Myosin VIIa (Proteus BioSciences, USA, 138-1), mouse anti-CtBP2 IgG1 (BD Biosciences, USA, 612044), mouse anti-GluR2 IgG2 (Merck-Millipore, Germany, MAB397), rabbit polyclonal anti-PMCA1 (Alomone labs, Israel, ACP-005), rabbit polyclonal anti-SERCA3 (Alomone labs, Israel, ACP-014), rabbit polyclonal anti-NCX (Alomone labs, Israel, ANX-011), rabbit polyclonal anti-MCU (Cell Signaling Technology, USA, 14997). The secondary antibodies used were as follows: Alexa Fluor 568-conjugated goat anti-mouse IgG1 (Invitrogen, USA, A-21124), Alexa Fluor 647-conjugated goat anti-mouse IgG2 (Invitrogen, USA, A-21235), Alexa Fluor 488-conjugated goat anti-rabbit IgG (Invitrogen, USA, A-11008), and Alexa Fluor 647-conjugated Phalloidin (Invitrogen, USA, A22287CN). Confocal images were acquired using a Zeiss LSM 880 with a 40 or 63×, 1.4 numerical aperture (NA) oil objective lens for Airscan imaging. The optimal voxel size was 0.1 μm along the x- and y-axes and 0.38 μm on the z-axis. For the immunofluorescence quantification, the fluorescence intensity was averaged in Gaussian volumes with standard deviations of 1 μm along the X, Y, and Z axes using Imaris software (Bitplane, Switzerland).

### Patch-clamp recordings from IHCs and two-photon calcium imaging

Patch-clamp recordings were performed using an EPC10/2 amplifier (HEKA Electronics, Germany) driven by Patchmaster software (HEKA Electronics, Germany). The extracellular solution containing: 110 mM sodium chloride, 2.8 mM potassium chloride, 25 mM tetraethylammonium chloride, 5 mM calcium chloride, 1 mM magnesium chloride, 2 mM sodium pyruvate, 5.6 mM D-glucose and 10 mM 4-(2-hydroxyethyl)-1-piperazineethanesulfonic acid (300 mOsm, pH 7.40), and the intracellular solution containing: 120 mM cesium methanesulfonate, 10 mM cesium chloride, 10 mM 4-(2-hydroxyethyl)-1-piperazineethanesulfonic acid, 10 mM tetraethylammonium chloride, 1 mM ethylene glycol-bis (β-aminoethyl ether)-N,N,N′,N′-tetraacetic acid (EGTA), 3 mM adenosine triphosphate magnesium, and 0.5 mM guanosine 5′-triphosphate sodium salt hydrate (pH ~ 7.30, 290 ~ mOsm). The patch pipettes (World Precision Instruments, USA) were pulled to obtain a resistance range of 5–6 mΩ. Recordings were discarded if the leak current exceeded −50 pA at a − 90 mV holding potential. All patch-clamp experiments were performed at room temperature, and the liquid junction potential was corrected offline.

### Noise analysis on Ca^2+^ tail currents

Nonstationary noise analysis of Ca^2+^ currents was used to estimate the number of Ca^2+^ channels per IHCs and their single-channel current [33]. We added 10 μm 1,4-dihydro-2,6-dimethyl-5-nitro-4-[2-(trifluoromethyl)phenyl]-3-pyridinecarboxylic acid methyl ester (BayK 8644), in the extracellular solution to maximize the open probability of the Ca^2+^ channels [86]. Hair cells were first held at −90 mV and then stepped to −110 mV, followed by a step depolarization to +50 mV and then stepped to −90 mV. As a result, the Ca^2+^ tail current was elicited and was recorded 100 traces for each IHC. The mean and variance of these currents were calculated point-by-point using a custom-made IGOR Pro analysis program. After plotting the variance against the mean current, the data were fit to a parabolic function: Var(I) = i · I − I2/N_Ca_ + E_noise_, where I is the mean current, i is the single-channel current, Enoise is the electrical noise, and N_Ca_ is the number of Ca^2+^ channels.

Whole-cell membrane capacitance (Cm) measurements in IHCs were performed with the lock-in feature and the “Sine+DC” method in Patchmaster software as our previous experiments. The increased Cm (ΔCm) after membrane depolarization was used to monitor exocytosis of IHCs, and the Ca^2+^ charge (Q_Ca_) was calculated by taking the integral of the leak-subtracted current during depolarization.

For calcium imaging, cells were loaded with Ca^2+^-indicator F4-FF (Thermo Fisher Scientific, USA) and Cy3-conjugated Ribeye-binding peptide (AnaSpec, USA), and then, two-photon microscope system (Scientifica Ltd., UK) were used to acquire intracellular Ca^2+^ signal (excited by ultrafast pulsed titanium–sapphire laser (Coherent Inc., USA) of 740 nm wavelength) using two-photon line scans (1.0 kHz) across the center of the fluorescent-labeled ribbon.

Ca^2+^ signals were measured as relative changes of fluorescence emission intensity (ΔF/F0). ΔF = F – F0, where F is fluorescence at time t and F0 is the fluorescence at the onset of the recording. The decay time of Ca^2+^ current transients was measured by fitting the calcium fluorescence decay with the following equation to assess the kinetic properties of Ca^2+^ clerance.

$${\rm{F}}={\rm{F}}0+{\rm{A}}1\exp \{-({\rm{t}}-{\rm{xt}}){\rm{\tau }}1\}+{\rm{A}}2\exp \{-({\rm{t}}-{\rm{t}}0){\rm{\tau }}2\}$$

where F0 is the initial luminescence intensity, A1 and A2 are pre-exponential factors, and (t–t0) is the difference between the initial time of measurement after excitation pulse t0 and time t. τ1 and τ2 is the fast and slow decaying component, respectively.

### Generation of hair cell conditional knockout mice

The *Pmca1*^*Loxp/+*^ mice were generated using the CRISPR/Cas9 system (GemPharmatech, China). Briefly, Cas9 mRNA, single guide RNAs and donor were co-injected into the zygotes, directing Cas9 endonuclease cleavage and Loxp site insertion in intron 8 and intron 10 of mouse *Pmca1* (NM_080636.2). As the *Pmca1* knockout (KO) mice die perinatally, the *Pmca1*^*Loxp/Loxp*^; *Gfi1*^*Cre/+*^ mice (*Pmca1* CKO mice) were used to specifically knockout PMCA1 in hair cells. *Pmca1*^*Loxp/Loxp*^;*Gfi1*^*Cre/+*^mice were confirmed by sequencing the mouse tail genomic DNA with the following primers. *Pmca1*^*Loxp/Loxp*^: 5’-TTGACCTGTCTTCCTAACG-3’ and 5’- ACCCACTGCAACTCTGTAAT-3’, *Gfi1*^*Cre/+*^: 5′-GGGATAACGGACCAGTTG-3′ and 5′-GCCCAAATGTTGCTGGATAGT-3′.

### Western blotting

The cochleae of both sides were quickly removed from the skull of mice and dissected in ice-cold PBS. For each sample, tissues of sensory epithelia (without spiral ganglion neurons) from 3 mice (6 cochleae) were mixed with ice-cold RIPA lysis buffer plus protease inhibitor cocktail (Thermo Fisher Scientific, USA) and phosphatase inhibitors. The samples were then centrifuged at 10,000 × *g* at 4 °C for 10 min, and the supernatants were collected. The protein concentration was measured by using a BCA Protein Assay Kit (Beyotime, China). The samples were added with 5×SDS sample loading buffer and boiled for 5 min. Protein samples were fractionated by polyacrylamide gel electrophoresis (PAGE) and blotted onto a Polyvinylidene Fluoride (PVDF) membrane. The membranes were blocked with blocking buffer (Beyotime, China) for 1 h at room temperature and then incubated with the primary antibodies including anti-PMCA1 Rabbit mAb (Alomone labs, Israel, ACP-005) at 1:1000, anti-cleaved caspase-3 Rabbit mAb (Cell Signaling Technology, USA, 9661) at 1:500, anti-Bcl2 Rabbit mAb (Cell Signaling Technology, USA, 3498) at 1:1000, anti-Cytochrome C Rabbit mAb (Cell Signaling Technology, USA, 4272) at 1:1000, anti-Bax Rabbit mAb (Cell Signaling Technology, USA, 2772), anti-Phospho-Histone H2A.X Rabbit mAb (Cell Signaling Technology, USA, 9718) at 1:500, anti-PARP1 Rabbit mAb (Abcam, UK, ab191217) at 1:500, and anti-beta-Actin Mouse mAb (Abcam, UK, ab6276) at 1:1000 overnight at 4 °C. The membranes were washed three times in TBS with Tween 20 buffer and then incubated with secondary antibody conjugated with horseradish peroxidase for 2 h at room temperature. After washes in TBS with Tween 20 buffer, membranes were incubation with the corresponding secondary antibody conjugated with horseradish peroxidase for 2 h at room temperature. The protein bands were detected by using an Amersham Imager 600 (G.E. Healthcare, Little Chalfont, UK). The Image J software was used to calculate the relative density of probe protein.

### Bulk RNA sequencing and analysis

Total RNA extracted from P12, P18, and P24 mouse cochleae was used for library construction and deep sequencing on the BGISEQ-500 platform (BGI-Shenzhen, China). Clean reads were mapped to the mouse reference genome (mm10) using STAR (v2.7.9a) with the following parameters: ‘--outFilterMultimapNmax 1 --outFilterIntronMotifs RemoveNoncanonical --outFilterMismatchNmax 5 --alignSJDBoverhangMin 6 --alignSJoverhangMin 6 --outFilterType BySJout --alignIntronMin 25 --alignIntronMax 1000000 --outSAMstrandField intronMotif --outSAMunmapped Within --outStd SAM --alignMatesGapMax 1000000’. We performed DEG analysis using the FindMarkers or FindAllMarkers function in Seurat (version 4.3.0). The DEGs were defined as genes with a fold change > 0.1 and adjusted *p* ≤ 0.05. In addition, the normalized data of the remaining genes was then Z-score transformed before executing the c-means fuzzy clustering of time-course regeneration data, with two centers and a cluster membership threshold of 0.8 [87].

### Single cell RNA-seq of IHCs

Prior to the harvesting procedure, all work surfaces were thoroughly cleaned with DNA-OFF (Takara Cat. #9036) and RNase Zap (Life Technologies Cat. #AM9780). Patch-clamp pipettes were pulled to an impedance of 3–4 MΩ resistance and filled with RNase-free intracellular solution containing: 125 mM potassium gluconate, 10 mM NaCl, 10 mM KCl, 1 mM EGTA, 2 mM MgCl2, 10 mM HEPES, 4 mM MgATP, 0.5 mM Na2GTP, 5 mM Na2Phosphocreatine and 1 U/µl recombinant RNase inhibitor (Takara Cat.no.2313 A) (pH 7.2, ~290 mOsm). RNA was collected by applying light suction until the cell had visibly shrunken. The contents of the pipette were ejected using positive pressure into an RNase-free PCR tube containing 5 μl of RNase-free lysis buffer and frozen. The lysate samples were vortexed and centrifuged, and placed on a preheated PCR machine for heat-treatment. Then, Smart RT buffer and RT enzyme were added, and the reverse transcription reaction was carried out according to the program. Add Smart Amp buffer-1 and Smart Amp enzyme-1 to the cDNA product obtained in the previous step and carry out the amplification reaction according to the procedure. Quantitative purification of cDNA was performed using a DNA purification kit. After Tn5 reaction, mix Smart Amp buffer-2, Smart Amp enzyme-2, S5XX, N7XX for library construction, purify and send for testing. Illumina NovaSeq 6000 sequencing platform was usually used, and PE150 sequencing mode was adopted. The recommended sequencing volume gene is 9 G, and additional sequencing volume can be performed according to actual requirements. Use FastQC software to evaluate the quality of the original data; then use HISAT2 software to compare the filtered data to the reference genome; then use Stringtie to calculate the gene expression and normalize it to generate the gene expression normalization file required for subsequent analysis; Gene expression standard files were used for further principal component analysis of samples, and software such as edgeR and DEseq2 were used to screen differentially expressed genes between groups, and GO, KEGG and Reactome enrichment analysis of differential genes was performed.

## Data analysis

GraphPad Prism 8.0, ImageJ, Igor Pro 7 (Wavemetrics), and R 4.2.1 (https://www.r-project.org/) were used for statistical analysis. Data normality was evaluated using the D’Agostino-Pearson and Shapiro-Wilk tests. Depending on the nature of the dataset, statistical significance was assessed using a two-tailed unpaired Student’s *t* test (normal distribution), Mann-Whitney *U* test (non-normal distribution), one-way/two-way ANOVA, followed by a Bonferroni post hoc test (normal distribution) and Kruskal-Wallis test (non-normal distribution). Results are shown as mean ± SD, and the level of significance was set to *p* < 0.05. Significant differences were reported as *p < 0.05, **p < 0.01, and ***p < 0.001.

## Supplementary information

Supplemental figure legends

Supplemental Figure 1

Supplemental Figure 2

Supplemental Figure 3

Supplemental Figure 4

Supplemental Figure 5

Related Manuscript File

## Source data

Source data

## Data Availability

The raw data of scRNA-seq have been deposited in the NCBI Sequence Read Archive (SRA) under accession BioProject codes: PRJNA1210011 (BioSample: SAMN46237090). The datasets used and/or analyzed during the current study are available from the corresponding authors upon reasonable request.
