# Supplementary material for: Folic acid prevents inner hair cell degeneration via genomic stability
Source: Cell Death Discov. 2025 Dec 2;12:31. doi: 10.1038/s41420-025-02880-4 (PMC12811298; doi:10.1038/s41420-025-02880-4)
Supplement: Supplementary file 1 — Supplemental figure legends [file 41420_2025_2880_MOESM1_ESM.docx]

**Supplemental Figure 1. Calcium influx and efflux in low-and high-frequency IHCs.**

A. Sketch of a mouse cochlear whole-mount that illustrates the positions of low-frequency and high-frequency region IHCs. Blue box: low-frequency; orange box: high-frequency `.

B. The number of Ca^2+^ channels per IHC was estimated. The hair cell was held at −80 mV, briefly hyperpolarized to −110 mV to relieve any steady-state inactivation, and then depolarized to +20 mV for 10 ms to open all Ca^2+^ channels. A Ca^2+^ tail current (lower trace) was elicited by repolarizing the hair cell from +20 mV to −80 mV (upper trace) in the presence of the L-type Ca^2+^ channel agonist BayK 8644 (10 μM). The decay of the tail current between the two dashed lines was used for noise analysis (upper). Here we show 100 superimposed traces (black) that were obtained with the same protocol. The mean (in back) and variance (in red) were calculated on a point-by-point basis (middle). The variance was plotted against the mean (in black) (lower). The number of Ca^2+^ channels, the single channel current, and open probability was estimated by fitting the data to a parabolic function.

C. Ca^2+^ current in inner hair cells (IHCs) from low-frequency and high-frequency region IHCs with Bay K 8644. The current amplitude (I_Ca_) was quantified.

D-E. Representative ΔC_m_ traces from double-pulse stimulation (two 20 ms depolarizations) in 10 mM EGTA, without (D) and with calcium extrusion channel blockers (E). The ratio ΔC_m2_/ΔC_m1_ quantifies replenishment capacity.

F. Confocal images of auditory hair cells from different turns of IHCs immunolabeled for CtBP2 (red), GluR2 (green) and Myosin VIIa(blue).

Statistical analysis by two-side unpaired *t* test or Mann-Whitney test with significance indicated and one-way ANOVA followed by the Bonferroni post hoc test with significance indicated. All data, the number of data, statistical test used and *p* values can be found in the source data file. N.S., not significant, **p*< 0.05; ***p* < 0.01; ****p* < 0.001.

**Supplemental Figure 2. The expression of NCX, SERCA and MCU along the cochlea.**

A. Confocal images of auditory hair cells from low- and high-frequence of basal membrane immunolabeled for NCX (green). CtBP2 was used to stain the presynapse (red).

B. Confocal images of auditory hair cells from low- and high-frequence of the basal membrane immunolabeled for SERCA3 (green). CtBP2 was used to stain the presynapse (red).

C. Confocal images of auditory hair cells from low- and high-frequence of basal membrane immunolabeled for MCU (green). CtBP2 was used to stain the presynapse (red).

Statistical analysis by two-side unpaired *t* test or Mann-Whitney test with significance indicated. All data, the number of data, statistical test used and *p* values can be found in the source data file. N.S., not significant, **p*< 0.05; ***p* < 0.01; ****p* < 0.001.

**Supplemental Figure 3. Stereocilia morphology in Pmca1 CKO mice.**

Confocal images of auditory inner hair cells (IHC) and outer hair cells (OHC) from wild-type mice (left) and *Pmca1* CKO mice (right) immunolabeled for stereocilia (actin) at P7.

**Supplemental Figure 4. Ribbon synapses and calcium influx in Pmca1 CKO mice.**

A–C. Confocal images of auditory hair cells from wild-type (left) and *Pmca1* CKO (right) mice at P12 (top), P18 (middle) and P24 (bottom), immunolabelled for CtBP2 (red), GluR2 (green) and Myosin VIIA (blue).

D–F. Representative traces of calcium ion currents from wild-type and *Pmca1* CKO mice at P12 (top), P18 (middle) and P24 (bottom), with current amplitude (ICa) quantified.

Statistical analysis by two-side unpaired *t* test or Mann-Whitney test with significance indicated. All data, the number of data, statistical test used and *p* values can be found in the source data file. N.S., not significant, **p*< 0.05; ***p* < 0.01; ****p* < 0.001.

**Supplemental Figure 5. Average expression of representative canonical marker genes.**

A-B. Heatmap showing the top 50 differentially expressed genes of IHCs between WT and *Pmca1* CKO mice at P12 (A) and P18 (B).

C. Average expression of *Otof*, *Slc17a8*, *Slc26a5*, *Slc26a4*, *Tectb*, and *Ucma* in harvested inner hair cells.
