# Supplementary figures and images for: Folic acid prevents inner hair cell degeneration via genomic stability
Source: Cell Death Discov. 2025 Dec 2;12:31. doi: 10.1038/s41420-025-02880-4 (PMC12811298; doi:10.1038/s41420-025-02880-4)

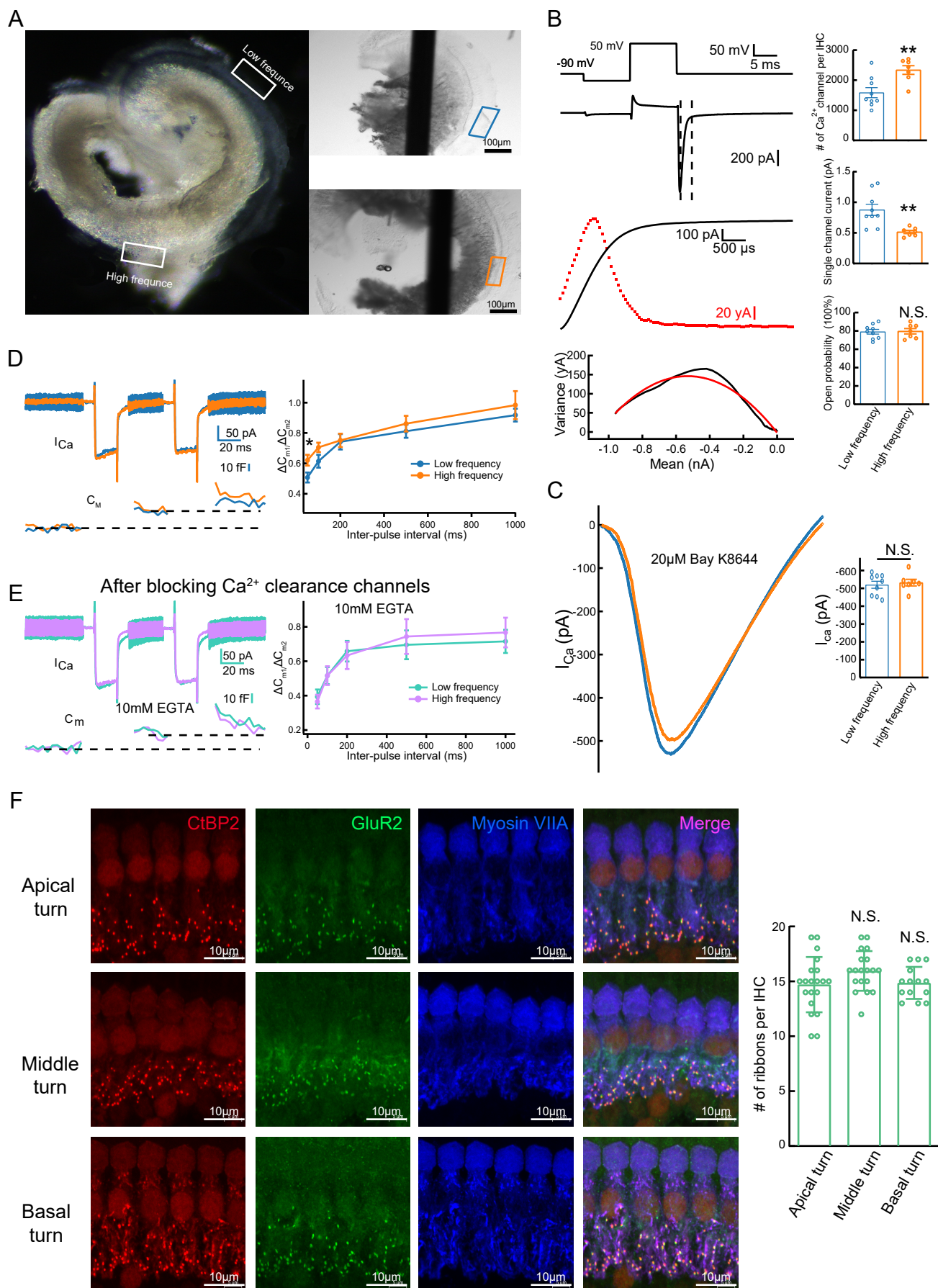

Supplement: Supplementary file 2 — Supplemental Figure 1 [file 41420_2025_2880_MOESM2_ESM.pdf]

A

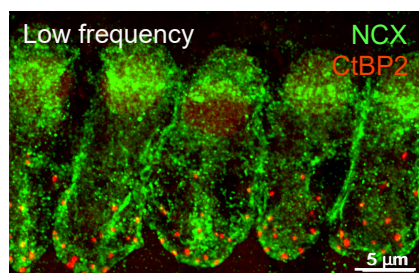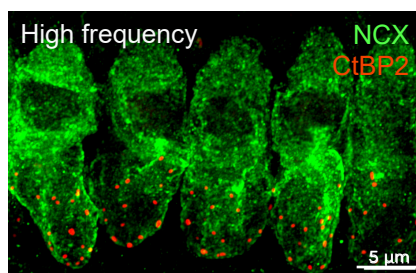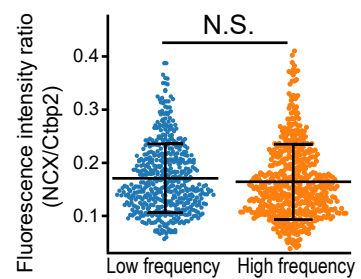

B

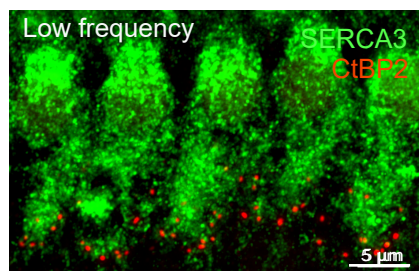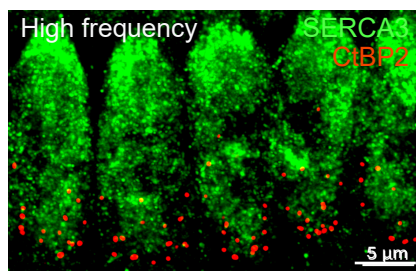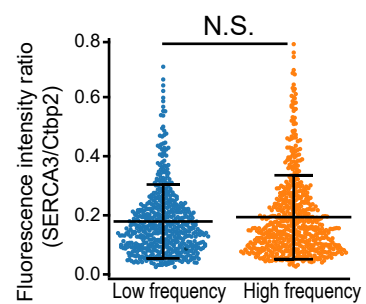

C

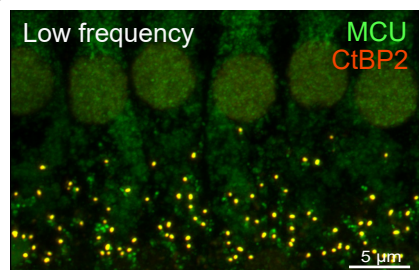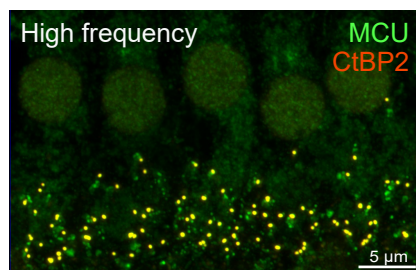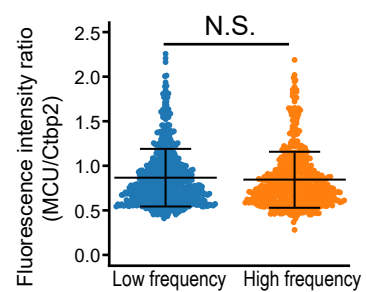

Supplement: Supplementary file 3 — Supplemental Figure 2 [file 41420_2025_2880_MOESM3_ESM.pdf]

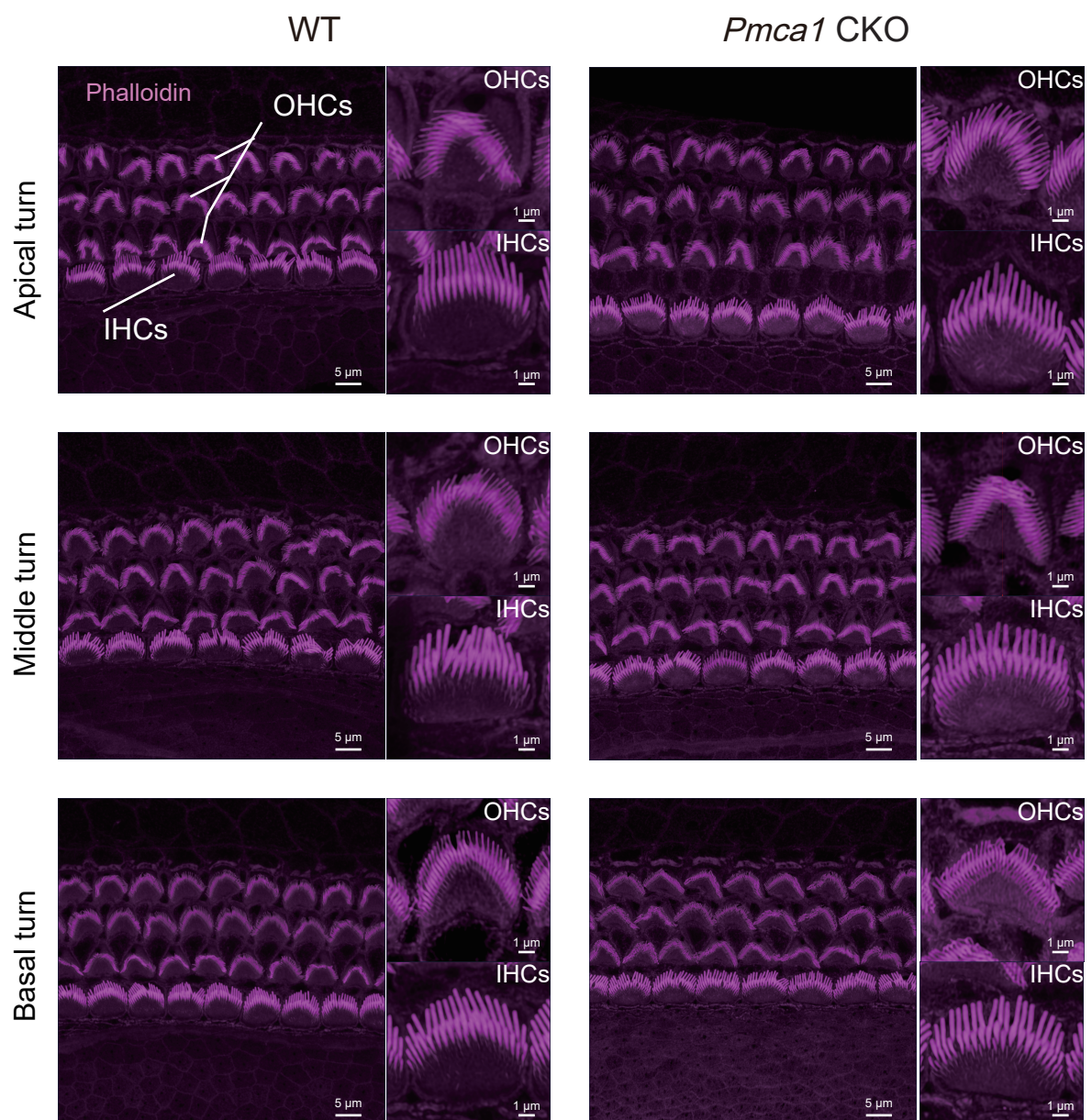

Supplement: Supplementary file 4 — Supplemental Figure 3 [file 41420_2025_2880_MOESM4_ESM.pdf]

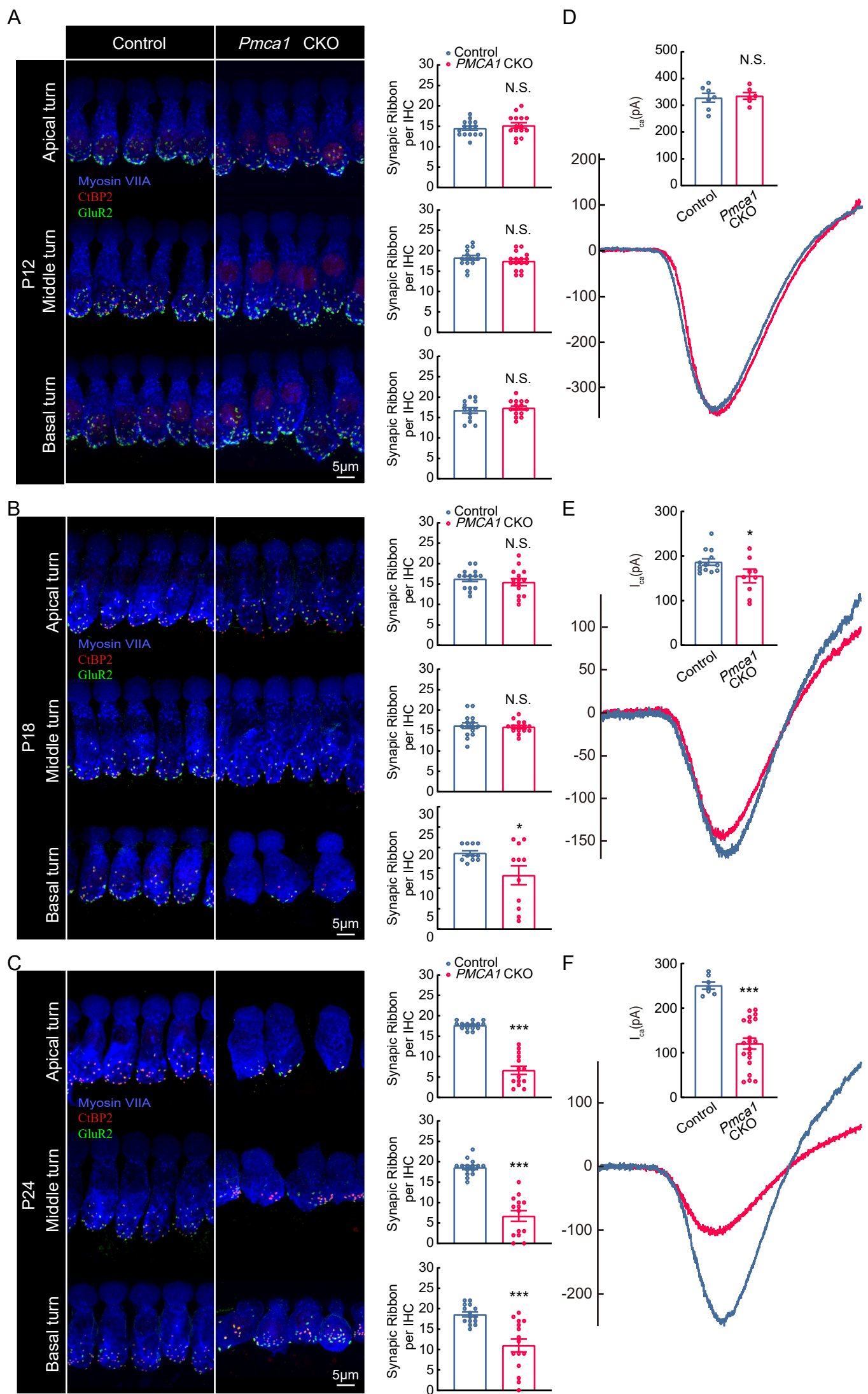

Supplement: Supplementary file 5 — Supplemental Figure 4 [file 41420_2025_2880_MOESM5_ESM.pdf]

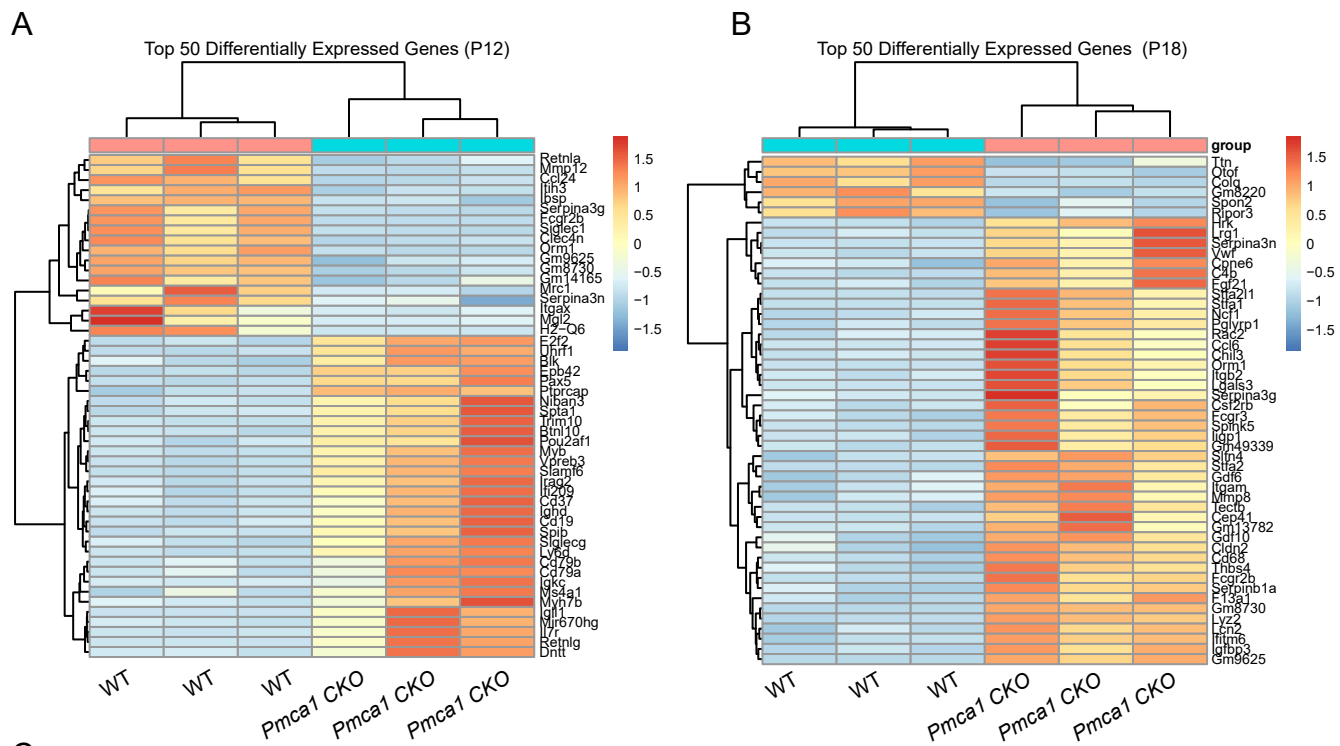

Supplement: Supplementary file 6 — Supplemental Figure 5 [file 41420_2025_2880_MOESM6_ESM.pdf]
