# Supplementary material for: Folic acid prevents inner hair cell degeneration via genomic stability
Source: Cell Death Discov. 2025 Dec 2;12:31. doi: 10.1038/s41420-025-02880-4 (PMC12811298; doi:10.1038/s41420-025-02880-4)
Supplement: Supplementary file 7 — Related Manuscript File [file 41420_2025_2880_MOESM7_ESM.docx]

**Folic acid could prevent calcium imbalance-induced hair cell degeneration by maintaining genomic stability**

**SUPLEMRNTAL MATERIALS**

**Western Blot Raw Data**
